# The Modulatory Effects of Non-Thermal Plasma on Seed’s Morphology, Germination and Genetics—A Review

**DOI:** 10.3390/plants11162181

**Published:** 2022-08-22

**Authors:** Livia-Ioana Leti, Ioana Cristina Gerber, Ilarion Mihaila, Paula-Maria Galan, Silvia Strajeru, Denisa-Elena Petrescu, Mirela-Mihaela Cimpeanu, Ionut Topala, Dragos-Lucian Gorgan

**Affiliations:** 1 Plant Genetic Resources Bank, 720224 Suceava, Romania; 2 Faculty of Biology, Alexandru Ioan Cuza University, 700505 Iasi, Romania; 3 Integrated Center of Environmental Science Studies in the North-Eastern Development Region, Alexandru Ioan Cuza University, 700506 Iasi, Romania

**Keywords:** non-thermal plasma, plasma treated water, seeds, gene expression, germination

## Abstract

Non-thermal plasma (NTP) is a novel and promising technique in the agricultural field that has the potential to improve vegetal material by modulating the expression of various genes involved in seed germination, plant immune response to abiotic stress, resistance to pathogens, and growth. Seeds are most frequently treated, in order to improve their ability to growth and evolve, but the whole plant can also be treated for a fast adaptive response to stress factors (heat, cold, pathogens). This review focuses mainly on the application of NTP on seeds. Non-thermal plasma treated seeds present both external and internal changes. The external ones include the alterations of seed coat to improve hydrophilicity and the internal ones refer to interfere with cellular processes that are later visible in metabolic and plant biology modifications. The usage of plasma aims to decrease the usage of fertilizers and pesticides in order to reduce the negative impact on natural ecosystem and to reduce the costs of production.

## 1. Introduction

According to the United Nations, the world population is expected to reach almost 10 billion in 2050 [1]. These data correlate with the results published by Food and Agriculture Organization about the acute food insecurity and the decrease of feedstock. Considering these facts, it is clear that the greatest challenge is to produce safe food of high quality, which can overcome individual nutritional intake for the population. The best approach is to improve agricultural productivity and reduce the pathogens that endanger seed’s viability. 

There has been an increased interest in finding an interdisciplinary solution to decontaminate the plant material and to enhance germination and development rate [2]. Cold plasma usage seems to satisfy all these conditions and it substitutes the usage of various chemical fertilizers that can affect human’s health. Moreover, the vegetal material can be exposed to plasma at any stage of development, seed, seedling or whole plant.

Researchers all over the world have developed a quite impressive database regarding plasma usage on natural (food [3,4,5], seeds [6,7,8,9,10], cells and tissues [11,12,13], wood [14,15]) and synthetic materials (polymers [16], fabrics [17,18], ceramics [19], metals [20], semiconductors [21]). It is essential to study the production sources of plasma, its parameters during treatment, and, subsequently, its effects on the chemical and physical structures of the treated samples. The effects vary in a dose-dependent manner and can be juggled based on the research goal.

Among the researchers that firstly studied plasma peculiar behavior we list the American scientist Irving Langmuir in the early 20th century [22]. Plasma is similar to a gas state, but it consists of a complex mixture of electrons, ions, photons, neutral atoms, radicals and molecules [23]. Still, plasma is electrically neutral due to the fact that the positive and negative charges are balanced overall. Plasma conducts electricity and responds to electric or magnetic fields [22]. It is thought that more than 99% of the visible universe is plasma, except cold celestial bodies and planets [23,24]. The stars, the solar winds, the northern and the southern lights are examples of natural plasmas found close to the humanity [25,26].

For plasma production, any gas can be exposed to high electric fields, high-energy radiation or high caloric energy [27] and all of this can produce ionizations of the gas atoms and/or molecules, resulting ions and electrons which generate stationary or transient plasmas [26,27]. Considering the temperature of the plasma components, one can separate thermal plasma (TP) from non-thermal plasma (NTP). Thermal plasma refers to a thermal equilibrium, which suggests the same value for temperature for all species, heavy particles (atoms, molecules or ions) and electrons [27]. Some general considerations about TP describe high electron densities and temperatures that reach millions of degrees (10^7^–10^9^ °C) [22]. Thus, it cannot be directly used in biological applications, save for the destruction of biohazard materials.

NTP is type of plasma that does not reach a thermodynamic equilibrium. Neutral particles and ions from the plasma have low temperatures, close to room temperature, while electrons have usually few orders of magnitude higher values of temperature. NTP associated with low degree of ionization and electron density [24,27]. The NTP sources can be tuned in order to not produce thermal damage for biological targets (in literature this plasma sources are known as cold plasma sources), and thus can be used in both biological and medical research [28]. This study focuses on non-thermal plasma and its effects on seeds.

Even though the experimental setups can vary based on the purpose of research and the laboratory equipment, there are few techniques which are usually used in agricultural research in order to produce NTP: atmospheric pressure plasma sources (jets, dielectric barrier discharges (DBD), corona and spark discharges, torches) and low-pressure plasma sources (radio-frequency discharges, direct current discharges). Dielectric barrier discharge is widely reported in scientific articles as the most common type of plasma production. This technique occurs when the electrical discharge is created between two electrodes separated by an insulating material and the temperature is close to the environment one [28].

## 2. Plasma-Driven Chemistry

In the case of low temperature plasma sources used for agricultural and life science applications, the plasma-driven chemistry is mainly responsible for the observed biological effects [29,30,31,32,33,34]. Biologically compatible plasma sources operating at atmospheric pressure are ignited using pulsed power sources (either alternative current sources or, more commonly, frequency dependent direct current pulses). The spatial and temporal behaviour of pulsed plasmas is essential to monitor and control during exposure in order to achieve a standardized set of exposure parameters for seed treatment. Either in direct plasma contact with specimens and liquids or during indirect exposure (specimen or liquid exposed to plasma gas exhaust), there is a limited number of regions where chemical reactions can occur at various rates, depending on the plasma source, working gas and pressure, target type, environmental humidity: the core plasma region, the plasma–air interface, the plasma-liquid interface, the core seed/liquid region [35,36,37].

In the core plasma region, as well at interfaces, electron collisions, electron recombination and Penning ionization processes of molecular species will lead to formation of short-lived and long-lived reactive chemical species such as atomic oxygen (O), singlet oxygen (^1^O_2_), atomic hydrogen (H), nitric oxide (NO•), nitrous oxide (N_2_O•), nitrogen oxides (NO_2_•, NO_3_•), ozone (O_3_), hydroxyl (OH•), hydrogen peroxide (H_2_O_2_), nitrogen acids (HNO_2_•, HNO_3_•). The rate coefficients of all processes leading to reactive chemical species formation are influenced by fundamental plasma parameters (e.g., reduced electric field, electron temperature, gas temperature). The presence of air impurities and environmental humidity also affects the concentration and accumulation of active species in plasma reactors for seed treatment [38,39].

Using a quadrupole-based mass spectrometer and quartz capillary, Dufour et al. [40] has sampled the gaseous phase from a seed-packed dielectric barrier device (6 kV, 600 Hz), fed with helium and N_2_ or O_2_, used for treatment of lentil seeds. The following reactive species were detected, with an increasing concentration as a function of reactive gas flow rate in admixture: O•, NO•, O_3_. Short lifetime species, such as OH• radicals, while expected to be present in the gas phase, will rapidly recombine and are difficult to be identified using capillary introduction mass spectrometry. This drawback is overcome using optical emission spectroscopy, and the spectral fingerprints of NO• and OH• radicals, as well as H or O atoms, can be easily identified in core plasma regions [40]. Molina et al. [41] proved that during plasma exposure of wheat seeds, dissociation of water molecules is predominating over processes involving air impurities, resulting in higher intensities of OH bands. The fingerprints of NO•, OH and O• were also detected by Nishime et al. [42] using a coaxial dielectric barrier discharge reactor used for treatment of winter wheat seeds, Rahman et al. [43] using a low pressure dielectric barrier discharge in Ar/O_2_ and Ar/Air to treat wheat seeds, Rongsangchaicharean et al. [44] using a streamer corona plasma (SCP) and dielectric barrier discharge (DBD) to treat rice seeds, Adhikari et al. [45] using a cold plasma air-jet instrument to prime tomato seedlings, Sarinont et al. [46] using a scalable dielectric barrier discharge device to treat seeds of radish sprouts, Gao et al. [47] using a dielectric barrier discharge plasma reactor to treat pea seed or tap water, Guragain et al. [48] using a gliding arc discharge reactor used to treat water, and Billah et al. [49] using a dielectric barrier discharge air plasma.

The stable molecules in the outlet of plasma reactors used for seeds treatment or for water treatment can be also analysed and quantified using Fourier transform infrared spectroscopy (FTIR) or ozone analyzers. Sivachandiran and Khacef [9] studied a cylindrical double dielectric barrier discharge reactor and a plate-to-plate double dielectric barrier discharge reactors used for treatment of radish, tomato and sweet pepper (Capsicum annum) seeds or water treatment and identified. FTIR spectra contain fingerprints of N_2_O• (30 ppm concentration), CO_2_, and O_3_ (850 ppm concentration). Rusu et al. [50] used an air dielectric barrier discharge to treat wheat seeds and a dynamic FTIR study (up to 6 min) of the gas phase indicates that the main stable products in the gas phase are O_3_ and N_2_O•. Machala et al. [51] studied DC-driven streamer corona and transient spark discharges and used electrochemical gas sensors, a gas analyser, and FTIR to monitor the concentration of NO• (up to 2000 ppm), NO_2_• (up to 400 ppm), and O_3_ (up to 100 ppm). Tomeková et al. [52] used a diffuse coplanar surface barrier discharge to treat dried pea seeds and the FTIR technique allowed them to identify the gaseous products of plasma: NO_2_•, N_2_O•, HNO_2_• and NO•.

Concerning the core liquid region, the dissolution of nitrogen oxides and nitrogen acids is leading to nitrites and nitrates, as well the acidification of unbuffered liquids. Transport from plasma core region to liquid core region is dependent on species-specific Henry’s law solubility coefficients. Many observations focused on H_2_O_2_, NO_3_^−^, and NO_2_^−^• species identification and quantification using spectrophotometric techniques. A series of plasma sources (i.e., transient spark, discharge with water electrospray and glow discharge with water cathode) were used by Yemeli et al. [53] to treat water and to study the effects of plasma treated water on maize and barley seedlings and by Kostoláni et al. [54] to study the effects of plasma treated water on dried barley grains and pea seeds. The H_2_O_2_, NO_3_^−^•, and NO_2_^−^• concentrations were found in the range 0.3–2.5 mM. Using an atmospheric pressure air DC discharge Xu et al. [55] pointed a linear increase of concentration as function of plasma treatment time up to 1.7 mM for H_2_O_2_ and 1 mM for NO_3_^−^• and NO_2_^−^•. Liu et al. [7] used a dielectric barrier discharge reactor in air, N_2_ or O_2_ to treat tap water or seeds (radish, mung bean, wheat, tomato, lettuce, mustard, Dianthus and sticky bean). Up to 120 min treatment time, the maximum values of concentration in treated water were found around 110 mg/L for NO_3_^−^NO^−^_3_, 30 mg/L for NO_2_^−^•, and 5 mg/L for H_2_O_2_. Judee et al. [56] quantified and monitored 16 long-lived species to characterize tap water treated using a peculiar dielectric barrier discharge and its effect on coral lentils seed. The study concluded that plant growth gain is linked to the effects on H_2_O_2_, NO_2_^−^•, NO_3_^−^•, NH_4_^+^, and HCO_3_^−^•, with the observed concentration up to 30 min treatment time being up to 3 mM/L. Machala et al. [57] measured for DC-driven streamer corona and transient spark discharges treated water concentration values for H_2_O_2_ up to 0.6 mM, NO_2_^−^• up to 0.7 mM and NO_3_^−^ up to 11 mM. Grainge et al. [58] used dielectric barrier discharge in air or He/O_2_ to treat water and the exposure time dependent concentration of H_2_O_2_ was reported up to 388 µM, OH• up to 80 µM, NO_3_^−^• up to 6 mM. Zhou et al. [59] used atmospheric-pressure air, O_2_, N_2_ and He microplasma arrays to treat aqueous solution containing mung bean seeds. The maximum concentration values as function of treatment time were observed for air plasmas: 18 mg/L for H_2_O_2_, 1.2 mg/L for NO_2_^−^• and NO_3_^−^•.

## 3. Effects of Non-Thermal Plasma Treatment on Seeds

**Figure 1:**
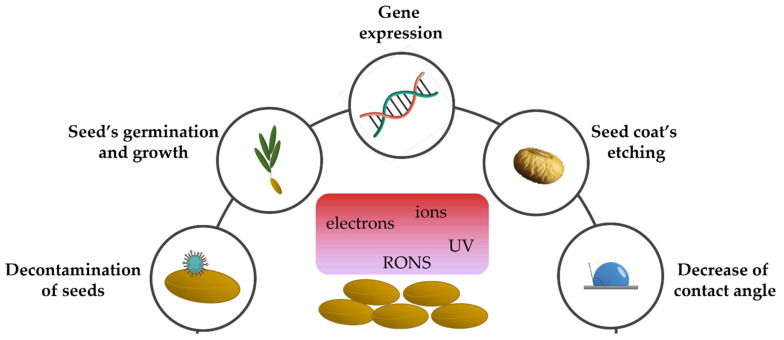
Cold plasma treatment on seeds influences decontamination, germination and growth processes, modulation of gene expression, seed coat’s etching and the decrease of contact angle.

Recent years have allowed researchers from different fields of interest to identify and understand the consequences of NTP treatment on various materials. Moreover, there has been increased interest in studying NTP treatment on various surfaces, both biotic and abiotic. RONS production is thought to have an important role regarding the beneficial effects of cold plasma exposure. An in-depth research on the scientific articles dealing with plasma showed that this type of treatment is mostly applied to seeds and food (Figure 1).

The effects of cold plasma on the various morphological, physiological, and molecular properties of the seeds is a poorly explored area of research, but with a great potential in terms of safer and more effective therapeutic approaches. In order to observe the evolution of researchers’ interest regarding the effects of plasma in agriculture, the scientific articles containing as keywords “cold plasma” and “seeds” were identified on the web search engine PubMed; the search was made on 27 June 2022. The selection depended on publication year of the articles and each article was individually verified in order to avoid confusions of terminology, such as the use of “plasma” as “plasma membrane” or “cold” as “cold resistance”.

**Figure 2:**
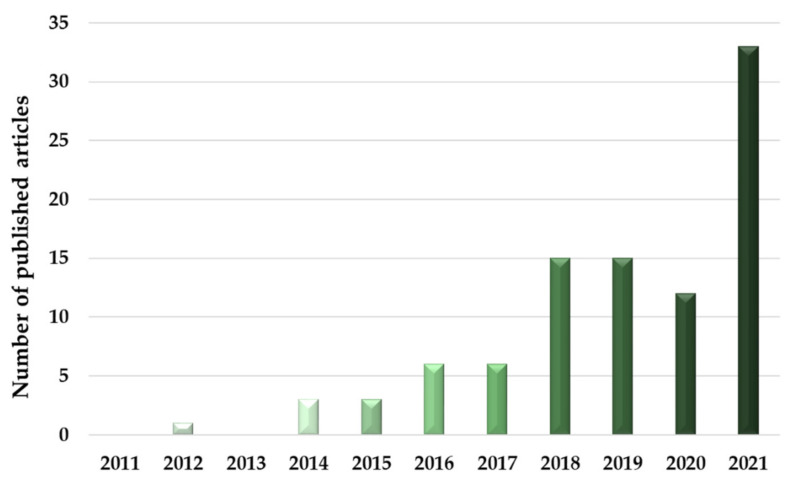
The number of published scientific articles about the cold plasma effects on seeds during 2011–2021 in PubMed.

The last four years showed, overall, a sharp increase in the number of published articles. These results strengthen the idea that cold plasma’s effects on seeds are still being discovered and each research paper enriches the total amount of information regarding this domain (Figure 2).

**Figure 3:**
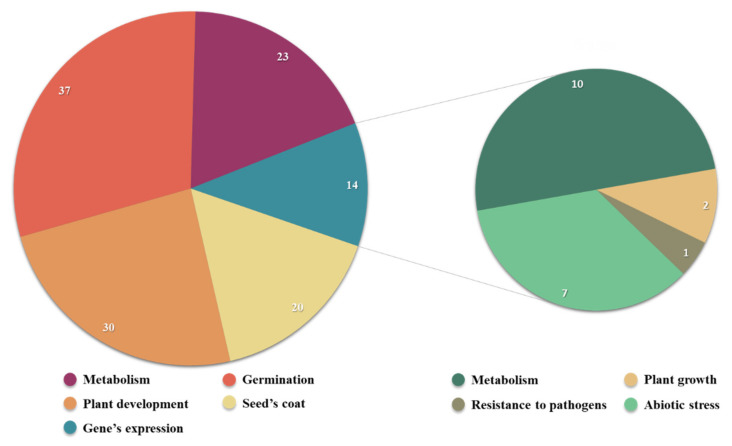
The frequency of the analyzed parameters in 50 scientific articles about cold plasma effects on seeds.

After a comprehensive research of 50 scientific articles regarding the effects of NTP treatment on seeds, the trend of the analyzed parameters is presented in Figure 3; one or more parameters can be mentioned in the same article. Thus, most studies focused on the modulation of germination process (37) and the further development of the plant (30). The researchers also studied the variation of multiple enzymes involved in various metabolic pathways (23) and the scarification of the outer layer of the seed (20). Out of 50 articles, only 14 traced the effects of NTP on the modulation of gene expression and most of them analyzed genes involved in the expression of enzymes involved in metabolic processes or in the plant’s response to abiotic factors. Only one study focused on the defense mechanisms against pathogen attack and one also analyzed the effects of plasma exposure on genes involved in the plant’s growth.

These results show that gene expression analysis after plasma treatment is a new approach for the researchers. The molecular analyses are still ongoing and there are still many cellular processes and interactions between molecules that must be understood.

### 3.1. Decontamination of Seeds

One important problem when it comes to seeds is the contamination with different pathogens, which endanger the germination and the development process. Plasma, due to the production of free radicals, is able to inactivate viruses [60] and a wide range of bacteria, such as spores [61,62] and biofilms [63,64]. In addition, Gram Positive and Gram Negative bacteria have a different sensitivity to cold plasma exposure caused by the variation of cell wall thickness [65]. Mravlje’s group [66] reported that the most common fungi that infect seeds belong to various genera, such as Alternaria, Aspergillus, Penicillium, Rhizopus, and Trichoderma.

All these results can be used in studies related to seed’s decontamination in order to identify the exact mechanisms by which all these microorganisms are destroyed. Machado-Moreira [10] suggested that seeds which will be consumed as germs can also be decontaminated using plasma due to the fact that it is a safe and efficient method.

Moreover, the effects of plasma were also studied on fruits and fruit juices. The results show that plasma modulates the amount of vitamins (A, B3, B6) [67,68], ascorbic acid [68], sugars [68], starch [69], carotenoids [70] and many others, in order to enhance the nutritional properties of the fruit juices. However, there are many concerns about how plasma influences food taste, flavor and aroma [3].

In terms of alcoholic drinks, red wine is a rich source of phenolic compounds, which have antioxidant properties and biogenic amines, compounds able to produce tachycardia and headaches. Niedzwiedz et al. [71] tested an alternative method of preservation and concluded that plasma treatment increases phenolic acid concentrations and decreases biogenic amines, results that favor the utilization of cold plasma as a novel sterilization technique.

The studies related to fruits or liquids (juices or wine) treatment show that cold plasma treatment is tested in various stages of plant development. However, the question is if an early seed stimulation can offer superior traits to the treated plants and if these effects are long-term. In addition to that, it would be interesting to determine if plasma treatment’s effects are specific depending on the plant’s stage of development or if these effects are common, regardless of the moment of exposure. Can plasma lower the amount of unwanted toxic compounds and increase the compounds which are useful for the body’s necessities only if it is applied on fruits, or is it possible to produce these consequences from the seed stage? Over the years, due to the enrichment of the scientific work related to plasma effects, we will be able to observe these aspects as well.

### 3.2. Seed’s Germination and Growth

Various approaches have been tested to improve growth process and defense mechanisms of plants. Non-thermal plasma might be an alternative to the conventional seed treatments, which include physical scratching, heat or chemical treatment [72]. Many studies in genetic engineering have been made [73], but there is a general concern regarding the safety of genetically modified organisms which restricts the use of this technique worldwide [74]. 

Other approaches focused on chemical treatments, but these are time-consuming, expensive and increase environmental pollution because of the noxious chemicals [75,76]. In the last years, physical treatments like the use of cold plasma gained attention, especially because this is an ecofriendly, easy handling and low cost strategy [77,78]. The plasma effects are highly complex, so it is necessary to adopt a systemic perspective, which forces researchers to first study the simple biomolecules and then complex tissues. Moreover, it is important to analyze the interaction between all reactive species found in plasma with different components of the cells in view of the complexity of both plasma and tissue [79,80].

One of the first studies to analyze the effect of plasma on seeds was published 1994 by Krapivina et al. [81] on soybeans. The results highlight an increase in germination and growth rate. Over the years, the research field expanded increasingly more and different seed species were exposed to various types of plasmas. Some of the studied species are cotton [82], coffee [83], grape [83], pea [84], wheat [85], soybean [86], sunflower [87], watermelon [88], corn [89], chicory [90], maize [91], thale cress [92], radish [93], rye [94], and zinnia [94].

Plasma treatment indirectly influences seed’s germination process through the production of various reactive species which provoke multiple changes in the biochemical profile. Reactive oxygen and nitrogen species interfere with abscisic acid and gibberellin pathways with effects on redox balance and breaking dormancy [38].

Overall, the results show that plants also produce endogenous RONS which have a dose-dependent effect [95]. High levels of RONS produce oxidation and affect plant growth [96], whereas low concentrations provoke dormancy breaking and encourage seed layers etching [97]. External addition of RONS might accelerate the first steps of germination [98,99].

One important factor in seed germination is dormancy breaking [100]. Dormant state characterize intact, viable seeds that do not complete germination process under favorable conditions [101]. After plasma treatment, UV radiation, chemical radicals production and chemical reactions induce changes on seed coat, stimulates rootlet generation, enhance root and shoot development and speed up the germination process [75,76,102]. In low concentrations, RONS like OH•, H_2_O_2_ and NO_2_• function as signaling molecules which modulate seed growth, development and defense against several factors [103]. 

The short-time plasma exposure of the seeds is a physiological process called priming and it is an ideal pre-treatment harmless for the seed, with pro-longed and stable consequences [104]. Short plasma exposure increases the length and the weight of the roots, while longer exposure inhibits plant growth [91].

### 3.3. Molecular Effects of Plasma Treatment in Plants

Guo et al. [105] used DBD in order to evaluate the germination process and the expression of 3 drought resistant related genes: LEA1, SnRK2 and P5CS. SnRK2 belongs to a serine/threonine kinase family which mediates the plant’s response to abiotic stress and abscisic acid-dependent plant development processes [106], while P5CS increases proline accumulation with beneficial effects on plant’s tolerance to stress [107]. Both genes showed an increased expression in plasma alone condition and plasma combined with drought stress. LEA proteins are involved in protecting plant from abiotic stress, such as cold, drought or salinity, but it also participates in plant’s normal growth and development [108]. The expression of LEA1 gene strongly decreased when wheat seeds were stimulated both with plasma and drought, which means that plasma stimulation does not interfere with LEA1′s stimulation pathways and this gene cannot offer an adaptive response in order to protect the plant during abiotic stress. Moreover, Guo et al. tested the gene expression 4 days after DBD exposure, which it is a relative short period of time to determine if plasma treatment is beneficial or not. Testing the molecular activity on long-term would be more relevant because in this way we would see the real effects of plasma treatment.

Iranbakhsh et al. [109] also published a study about plasma effect on *Triticum aestivum*. The seeds were directly exposed to DBD for different periods of time (0, 15, 30, 60, 90, 120 s) and at 3/6 h after treatment, RNA was isolated from root and shoot and HSFA4A gene expression was analyzed using quantitative Real Time-PCR (qRT-PCR). HSF (Heat Shock Factors) family represent transcriptional factors which mediate the activation of a wide set of stress-related genes [110]. HSFA4A was shown to function as a substrate of the MPK3/MPK6 signaling pathway which acts as a key regulator of plant immunity [110]. As for the root, the results show an increased expression of HSFA4A gene 3 h after the 15 s plasma treatment. In the shoot, increased expression of HSFA4Awas also observed after 6 h from the plasma treatment. These results show that the expression of HSFA4A in the root triggered more quickly than in the shoot, but in the shoot the expression levels are higher. We can see that plasma effects are not homogeneous throughout the plant and the expression of some genes can vary in different parts of the plant in terms of debut and duration of expression.

Therefore, both articles previously mentioned analyzed wheat, and in both cases, plasma was generated using the DBD method, but the exposure time varies from a few seconds to 2 min according to Iranbakhsh et al. up to 4 min in Guo et al. This aspect emphasizes that there is not a standardized protocol to treat the plant material, which makes it difficult to compare even seeds belonging to the same species, whether we talk about morphological or molecular aspects. It would be interesting to see the long-term effects of plasma exposure and to see if the results obtained right after exposure persist during plant’s growth.

The effect of NTP was analyzed even on plants contaminated with different pathogens. For instance, Panngom et al. [111] studied molecular aspects of *Solanul lycopersicum* seeds when infested with *Fusarium oxysporum* spores. The qRT-PCR analysis focused on pathogenesis related (PR) genes of the tomatoes. A total number of seven genes was analyzed and three of them (PR1a, PR1b, PR3a) showed an increased expression after 10 min of plasma treatment. Plasma generates various ROS and RNS which act as an activator for plant’s resistance mechanisms [112]. This study emphasizes the idea that plasma treatment enhanced the response of PR genes and plant’s health and growth were not affected.

Adhikari et al. [6] also analyzed the response of pathogenesis related genes of tomato seeds, but with an indirect plasma treatment. Plasma treated water (PTW) was generated using cold-atmospheric-air jet plasma and 10-days old tomato seedlings were treated for three different exposure times (10, 15 and 60 min). Real Time PCR analysis was made from leaves and roots. Best results were obtained from leaf samples registered for 60 min PTW, while in roots the lower treatment time most stimulated the interest genes. These molecular data of gene expression support the study of Shen et al. [113], who demonstrated the bactericidal effects of plasma treated water using scanning electron microscope analysis.

Therefore, many articles confirm the antimicrobial activity of plasma, even if it is a direct or indirect treatment. The results are valuable as they focus on different species (tomato/wheat), the plasma generation source varies, and the treatment technique is different (indirect/direct treatment).

Adhikari et al. [45] published another article where the stimulation of tomato seeds was made using cold plasma jet with an exposure of 1, 5 or 10 min. The molecular analysis included the evaluation of antioxidant, pathogen resistant and epigenetic regulation related genes. The expression of allene oxidase (AOX) and 12-oxo-PDA increased significantly after 10 min of plasma treatment. Most of redox homeostasis genes and pathogen resistant gene followed a similar pattern of expression and the most important activation occurred after 10 min of plasma treatment. 1/10 min treatment time also induced the expression of HAT (histone acetyltransferase) and HFMET (histone-lysine N-methyltransferases). Overall, the results suggest that 1- and 5-min treatments have more similar effects on gene expression than the 10-min one. This could mean that the chosen treatment times do not produce significant different results.

Zhang et al. [114] studied the effects of argon plasma on *Glycine max* sprout. The results show that plasma treatment upregulates different subunits of ATP (a1, a2, b1, b2, b3), target of rapamycin (TOR) and growth-regulating factor (GRF) 1–6 and decreases the level of expression for ATP MI25. This article proves the possibility of treating plants at any stage of their development and confirms plasma’s modulation effects on plant’s development due to the activation of metabolic related genes. This study also analyzes cytosine methylation, an important epigenetic mechanism that regulates gene expression. The results support the idea of enhanced germination as plasma acts even at epigenetic levels and produces demethylation of ATP, TOR and GRF.

In 2016, Ji et al. [2] applied high voltage nanosecond pulsed plasma and micro DBD plasma on *Spinacia oleracea* seeds. The authors prove that, in the moment of germination, there is an increase of starch degradation enzyme, which is essential for breaking down nutrients stored in seed’s endosperm. Other studies also show that the seed’s reserves influence the success of development of young seedlings as they are degraded by various enzymes and ensure the nutritional intake needed by the seed to grow [115]. The results show that only high voltage nanosecond pulsed plasma slightly increased pullulanase gene expression, but the molecular analysis was performed only once, one day after treatment. These results emphasize the large variability of plasma treatments, how different methods of plasma production or different exposure times can have multiple distinct effects and how important is to identify the proper treatment. Considering the results published by Ji et al., spinach seeds are not influenced by micro DBD plasma, and only short treatments with high voltage nanosecond pulsed plasma can easily increase germination process.

*Cannabis sativa* has some unique active secondary metabolites called cannabinoids, which are part of plant’s defense machinery [116]. Iranbakhsh et al. [77] studied both the expression of four genes involved in the production of four key enzymes involved in the biosynthesis of cannabinoids (CBDAS, THCAS, OASC and OLS) and WRKY transcription factors, which are known as significant regulatory genes. The WRKY gene family modulates a multitude of biological processes, like plant immunity [117], response to biotic and abiotic environmental stress [118], and senescence [119]. The plasma was produced using DBD for different exposure times (0, 40, and 80 s). The results for the genes involved in the production of cannabinoids show that the 40 s treatment enhanced mRNA expression, especially for OAC (42 folds), followed by OLS, THCAS, and the lowest increase was for CBDAS (19.5 folds). The transcription of WRKY was upregulated by 9.8 and 13.3 folds, respectively for the 40- and 80-s plasma treatment. The overexpression of these genes is correlated with beneficial effects on plant growth and development, but there is no doubt regarding a strong plant–plasma interplay. 

Suriyasak et al. [120] analyzed if the germination of heat stressed-rice seeds can be improved using plasma treatment by modulating epigenetic activity, such as DNA methylation. The results show that plasma caused methylation of the gene promoter involved in ABA biosynthesis, but it decreased the level of methylation in gene promoter responsible for ABA catabolism and α-amylase genes. Considering these results, the qRT-PCR data show a decrease of ABA biosynthesis genes expression and an increase for ABA catabolism and α-amylase genes, results that strengthen the information about the level of methylation. This article is of great interest considering the fact the condition of preheated seeds (the seeds were kept at 27 °C during germination) might be associated with global warming, which is an imminent environmental stress factor with terrible effects on crop productivity and sustainability.

For example, if we talk about DBD type of treatment, we can see from Table 1 that the exposure times vary from 3 s to several seconds (10 to 15) or even minutes (4 to 10). Moreover, two independent scientific research groups exposed wheat seeds to very different exposure times (15 s to 4 min), so there is a high variability regarding the experimental design, and it can differ within the same species.

For example, authors tend to test multiple plasma generation methods with various exposure times, which can vary from a few seconds [2,109,121,122] up to several minutes [2,6,45,105,111]. The molecular effects are usually tested a few hours or a few days after the moment of exposure and, overall, there seems to be a wide variation in the response of interest genes. Still, what happens to the plant weeks or even months after plasma exposure? Is plasma treatment able to increase the number of fruits produced per plant? Or do mature plants have a greater resistance to abiotic stress later in life? These are long-term effects which probably will be tested, but for now the scientific community is trying to identify short-term consequences and to assemble information regarding morphological, physiological, biochemical and molecular aspects in order to decide the further research directions. All these results and others are summarized in Table 1.

### 3.4. Seed Coat’s Etching and Hydrophilicity

Seed quality is a quite complex concept because it depends on many parameters, including the germination process. Germination started with the uptake of water by imbibition, followed by the emergence of the embryo [101]. However, the rigidity of the coat might delay this process as it may not allow the enough wetting of the seed [125].

The experimental design used in different studies starts with dry seeds exposed to plasma treated water, followed by morphological, physiological, and molecular evaluations during the germination process. Therefore, the water imbibition process is enhanced through the pores that are created in the seed coat as a consequence of plasma exposure.

Plasma treatment on seed coat affects both chemical structure and roughness of the surface [126]. Even though the results clearly show that plasma treatment enhances water absorption and germination, the precise mechanisms by which the seed coat becomes more permeable are unknown. One possible explanation could be the oxidation of seed’s top layers and insertion of some oxygen-containing functional groups [127,128,129]. Moreover, there might be chemical etching through oxidation [130] or even physical etching through ion impingement which provokes sputtering [131].

Viewed under the microscope, the outside layer appears as a wavy, but flat film. Ling et al. [86] and Meng et al. [128] sustained that great amounts of energetic ions and active species erode and etch the seed surface, creating holes, which allow water absorption. Among the effects produced by plasma on the seed coat are disruption, abrasion or even the loosening of original structures [125]. All these mechanisms cause changes of the hydrophobic wax layer of seed coat with a transition to a hydrophilic coat [132].

Li et al. [133] concluded that the DBD plasma treatment on wheat seeds increased water uptake to a certain limit, and then these values decreased with increasing plasma exposure time. Moreover, using scanning electron microscopy, some square mesh structures appeared on wheat’s seed coat, structures which were gradually destroyed after DBD plasma treatment. These results are correlated because they show that the change of seed coat morphology enhances the water permeability.

The plasma treatment effects depend on the voltage and time exposure. Tong et al. [104] established that the increase of these two parameters creates several tiny holes on the surface of the seeds. For instance, Dauwe et al. [134] exposed *Linum usitatissimum* seeds to cold plasma for 5/10/15/20 min and observed that the exposure which lasts 10 min or more creates too many and too large holes which negatively affect the seed instead of accelerating the germination process. The etching or the eroding effect on seed coat was also observed on barley [80], wheat [41,76,105,129], oat [76], bitterweed [104], lettuce [135], linseed [134], cotton [136], lentil [129], common bean [129], sunflower [137], artichoke [138], and Arabidopsis [92]. Moreover, surface etching had also been reported in studies which used plasma treatment on other samples such as wood [139] in order to improve wettability.

### 3.5. Decrease of Contact Angle

**Figure 4:**
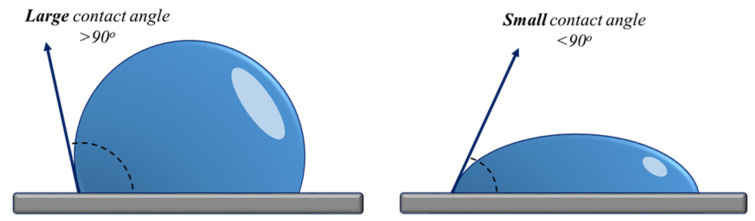
Contact angle measurement.

The contact angle is measured when a liquid-air interface meets a solid surface and quantifies the degree of wetting. Small contact angles (<90°) are correlated with increased wettability, whereas large contact angles (>90°) correspond to low wettability [140] (Figure 4). The decrease of contact angle is also associated with an increased hydrophilicity of the seed coat, which allows water to enter the seed much easier. The same effects are reported in Svubova’s paper [141], where soybean seeds showed an increased water uptake after cold atmospheric plasma treatment for different exposure times.

One method to decrease the contact angle is plasma treatment and some studies show that air plasma is more efficient than the nitrogen one [142]. Moreover, there are various plant species which reacted similar about the lessen of the contact angle, such as lentil [129], bean [129], wheat seeds [129], sunflower [137], artichoke [138], and mungbean [59]. Therefore, the contact angle reflects the hydrophilic ability of the seed, which is a key factor in an efficient germination process [86].

## 4. Plasma Treated Water

**Figure 5:**
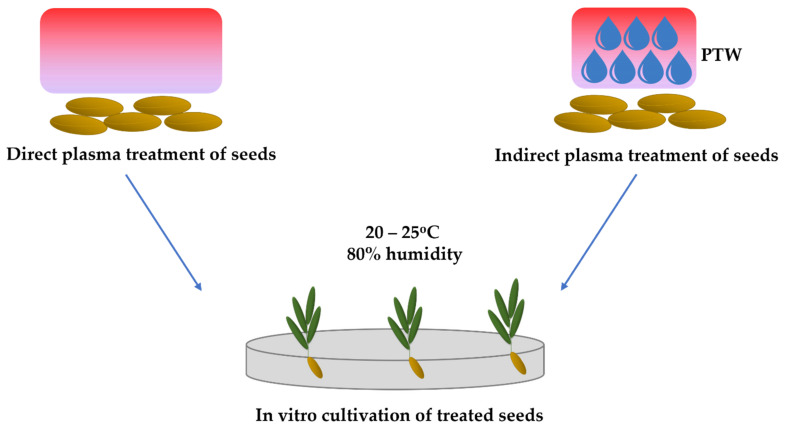
Direct (**left**) versus indirect (**right**) treatment of seeds using cold plasma.

Most studies found in literature focused on direct treatment, which consists in direct exposure of seeds to plasma. This technique presents advantages for some research fields, but indirect treatments have drawn attention lately and reveal new insights regarding the improvement of seed’s developmental processes. The approach implies exposing water to plasma, followed by its use for spraying or sinking the seeds (Figure 5). Plasma treatment of water is usually called plasma treated water (PTW) and it is characterized by various chemical and physical modifications which later induce different biological effects on food and seeds at different time scales [30,143]. PTW chemistry varies depending on the type of plasma source used, the working gas, the plasma source–water surface distance, and treatment time [7,29,56,57,144].

The modulation of PTW’s properties may refer to change of pH, redox potential, conductivity, as well as the content of reactive oxygen (ROS) and nitrogen species (RNS) [30]. The oxidation-reduction potential (ORP) of water indicates its disinfection potential [145]. PTW leads to microorganisms’ cell membrane damage due to a high oxidizing capacity [146]. Conductivity refers to water’s capacity to allow the electric current to flow through it. During the PTW generation, the action of electrical discharge includes the action of energetic electrons which will dissociate nitrogen and oxygen molecules, producing various solvated ions and radicals, like ozone and nitrogen oxides [9]. Conductivity depends on the reactive oxygen and nitrogen species (RONS) formed during plasma production. The ions produced enhance conductivity, which increases the radiated power [30].

In addition to that, there is a variation in the hydrogen ions number produced during plasma action which lowers the pH values [147]. The acidification of PTW is caused by the production of chemical species, mainly by nitric acid [148]. The pH changes in PTW vary depending on the reactor [149] and feed gas [29] used for plasma generation. 

Adhikari et al. [6] analyzed the pH variations and observed that CAP decreased the pH to more acidic values. Moreover, the H_2_O_2_ and NO• amounts were measured using a spectrophotometer and an increase in the concentration of these two chemical compounds were observed after plasma treatment. The various RONS formed in plasma treated water affect the endogenous RONS level with serious effects on plant’s metabolic responses. Similar results were also found by Mijia-Teniente et al. [150], Jiang et al. [96], and Guler and Pehlivan [151]. Several articles show that the accumulation of H^+^ ions has a negative effect on germination process [43].

Other studies show that after ten or five minutes of plasma exposure, the pH dropped from 7 to 3.2 [152], respectively 3.7 [153]. Both studies reported an increased conductivity and a decreased pH and these results suggest the presence of active ions in PTW [9]. Vlad and Anghel [154] support the idea that both parameters depend on nitric acid concentrations in water.

The long-term storage of PTW preserves the acidification produced during plasma generation [155]. The optimum pH of water for plant cultivation is 5.6–5.8 [147]. After 70 h of storage, there was a decrease from 2.07 to 1.5 [156] and after 30 days from 6.8 to 2.3, regardless of the storage temperatures [113]. Figueira et al. [143] observed that after 48–72 h of storage at 24 °C and 3 °C, pH returned to neutral values, due to short-lived reactive species that disappear after plasma generation. This observation needs to be correlated with the pH values obtained by Figuiera immediately after plasma stimulation because the lowest pH level was 3.5, which is higher than the values mentioned in the previous articles. The different results regarding the long-term storage effect on PTW’s pH can be attributed to the distinct plasma generation methods, considering the fact that from the beginning Figueira et al. obtained higher pH values. Therefore, perhaps gliding arc discharge method used by Figueira’s group produces more short-lived reactive species that disappear over time comparative to plasma microjet device used by Shen [113], which caused the pH to drop to almost 1 and remain at this level over time. Some researchers support the idea that, during plasma exposure, there are various short and long-lived reactive species that influence pH values [154]. Depending on the chosen setup, the concentrations of these compounds can vary and react differently to long-term storage.

The results also show that pH and conductivity of PTW depend on many parameters, such as treatment time, storage temperature and period, but also on the interaction of all these factors. Moreover, it is thought that positive temperatures induce important modifications in PTW over time [154]. This statement is supported by Shen et al. [113], who evaluated PTW antimicrobial activity after different storage temperatures and concluded that −80 °C conditions best preserved this characteristic, whereas 25 °C, 4 °C and −20 °C showed a decrease in antimicrobial action. Therefore, we can say that not pH itself determines the antimicrobial activity, but the reactive species which are formed during plasma exposure. As previously mentioned, RONS are thought to be the main chemical compounds with bactericide effect and we can conclude that they are better preserved at very low temperatures, like −80 °C.

Plasma generation implies the production of radiation that belongs to ultraviolet range. Under this radiation and free electrons, the water molecules in gas phase dissociate, regroup and form OH• radicals [147]. Moreover, H_2_O_2_ acts as a signaling molecule and is involved in breaking seed’s dormancy by activating various proteins/genes related to plant growth [9,157]. Meanwhile, results obtained by Mahanta et al. [158] show that plasma treated water also enhances germination, but further studies are necessary in order to optimize the experiment because treated seeds showed some modifications in the seed’s structures.

Atmospheric nitrogen and oxygen atoms form nitric oxide (NO), which will interact with OH radicals, oxygen, and water molecules, forming nitrite (NO_2_^−^•) and nitrate (NO_3_^−^•) [147]. It is thought that the increase of nitrite and nitrate ions might be the main factor which enhances plant growth [30]. Various studies showed that NO_3_^−^• represents an important source of nitrogen, as this chemical element is a part of many molecules, such as amino-acids, proteins, chlorophyll, and different cellular components [9].

Different RONS participate in plasma’s antibacterial activity, leading to membrane and cell wall damage, as well as DNA and protein degradation [159]. Some researchers prefer to keep PTW for one hour or one day at room temperature in order to reduce short lifetime RONS concentrations before treating the seeds [93].

So, the two methods of treatment (direct or indirect) have different consequences regarding the seed’s response, mainly due to the fact that the chemical substances which interact with the seeds are different. Direct exposure favors the formation of short-lived reactive species that affect seed’s coat, while these components no longer exist in PTW. PTW contains long-lived compounds or reaction products derived from the short-lived ones.

## 5. Conclusions

Plants are deeply affected by the continuous environmental changes, including extreme temperatures, as well as the attack of various pathogenic bacteria that have developed resistance to antibiotics and pesticide treatments. The constantly changing environment provoke plants to adapt and their response include changes in gene expression, metabolism, and physiology, with consequent effects on plant growth and development [160]. Recent studies focus on the replacement of chemical fertilizers and physical scarification with environmentally friendly techniques.

Literature data show that plasma treatment of plants can be a solution for decontamination, increased hydrophilicity, and stimulation of the expression of different genes related to embryonic development, plant growth, and resistance to pathogens. This approach has been tested on numerous species of plants and, overall, the results show the potential of creating stronger plants in terms of abiotic stress resistance and enhanced development. This is a great advantage of this technique because it shows its efficacy when testing different samples and it is proven that the beneficial effects are not isolated but can be applied to entire vegetal resources.

## Figures and Tables

**Table 1:** An overview of the non-thermal plasma effects on gene expression of various species of seeds.

Seed Type	Plasma Treatment Parameters	Plasma Effect on Gene Expression	Reference
*Triticum* *aestivum*	DBD 13.0 kV, 50 Hz 4 min	Increased expression for SNRK2 and P5CS with/without drought stress	[105]
		**LEA** gene expression is increased without drought stress and decreased when exposed to drought stress	
*Triticum* *aestivum*	DBD 80 W 15/20/60/90/120 s	The expression of **HSFA4A** is triggered more quickly in the root than in the shoot, but the expression levels are higher in the shoot	[109]
*Solanum* *lycopersicum*	DBD 0.75 kV, 7.5 W, 80 mA 10 min	Overexpression of **PR1a**, **PR1b** and **PR3a** (pathogenesis related genes)	[111]
*Solanum* *lycopersicum*	Cold atmospheric-air jet plasma 0.66 kV, 83.5 kHz, 70.39 mA 15/30/60 min	Increased expression of **mitogen activated protein kinase (MAPK) gene**	[6]
*Solanum* *lycoperiscum*	Cold plasma jet 0.68 kV, 83 kHz, 77 mA 1/5/10 min	After 10 min of plasma exposure, there was an increase of **AOX**, **12-oxo-PDA**, **HAT and HFMET gene expression**	[45]
*Glycine max*	Argon plasma 10.8–22.1 kV 1–2 min	Upregulation of **ATP subunits** (a1, a2, b1, b2, b3), **target of rapamycin (TOR)** and **growth-regulating factor (GRF)**	[114]
		Decrease **of ATP MI25** gene expression	
		Increases demethylation of **ATP**, **TOR** and **GRF**	
*Spinacia oleracea*	Nanosecond pulsed plasma 6–27 kV, 0.7–2.3 kA Micro DBD plasma 6 kV, 14 mA, 22 kHz For micro DBD: 30 s/1/3/5 min	Upregulation of **pullulanase** gene expression level when exposed to nanosecond pulsed plasma	[2]
*Cannabis* *sativa*	DBD 0.84 W cm^−2^ 40/80 s	The 40 s treatment time enhanced the expression of **OAC**, **OLS**, **THCAS**, **CBDA** and **WRKY** genes	[77]
*Oryza sativa*	DBD 7.96 kV, 9.2 kHz, 2.17 W Total time: 3 min (intermittently treated at 10/60 s)	Modulation of methylation level of promoters, which cause a decrease of ABA biosynthesis gene expression and an increase of ABA catabolism and α-amylase gene expression	[120]
*Andrographis paniculata*	DBD 9.7 kHz, 30 V, 2.4 A 3 s	Downregulation of **NCED5** gene	[121]
		Upregulation of **ACO**, **NRT1** and **PRP3** genes	
*Arabidopsis thaliana*	Radiofrequency plasma 20 Pa	The growth enhancement of seeds seems to be an epigenetic mechanism which is not passed to the next generation and does not involve changes in gene sequence	[123]
*Pisum sativum*	Diffuse Coplanar Surface Barrier Discharge 400 W 60–300 s	Using alkaline comet assay, it was shown that cold plasma decreases **DNA damage** in seeds treated from 120 to 240 s	[84]
*Zea mays*	Diffuse Coplanar Surface Barrier Discharge 400 W, 20 kV, 15 kHz 30/60/90/120/180/300 s	Upregulation of **HSP101** and **HSP70** genes	[122]
*Hordeum* *vulgare*	Diffuse Coplanar Surface Barrier Discharge Ambient air/oxygen/nitrogen 400 W, 20 kV, 15 kHz 10/20/30/60/180/300 s for each working gas	Using alkaline cornet assay, it was shown that ambient air and oxygen plasma caused an increase in DNA single/double strand breaks, while nitrogen plasma showed no damage	[124]

## Data Availability

Not applicable.
